# Maternal Diabetes and Postnatal High-Fat Diet on Pregnant Offspring

**DOI:** 10.3389/fcell.2022.818621

**Published:** 2022-05-30

**Authors:** Yuri Karen Sinzato, Verônyca Gonçalves Paula, Franciane Quintanilha Gallego, Rafaianne Q. Moraes-Souza, José Eduardo Corrente, Gustavo Tadeu Volpato, Débora Cristina Damasceno

**Affiliations:** ^1^ Laboratory of Experimental Research on Gynecology and Obstetrics, Postgraduate Course on Tocogynecology, Botucatu Medical School, Sao Paulo State University (UNESP), Botucatu, Brazil; ^2^ Research Support Office, Botucatu Medical School, Sao Paulo State University (UNESP), Botucatu, Brazil; ^3^ Laboratory of System Physiology and Reproductive Toxicology, Institute of Biological and Health Sciences, Federal University of Mato Grosso (UFMT), Barra do Garças, Brazil

**Keywords:** Female reproduction, diabetes, malnutrition, fetal programming, rodent

## Abstract

Maternal diabetes-induced fetal programming predisposes offspring to type 2 diabetes, cardiovascular disease, and obesity in adulthood. However, lifelong health and disease trajectories depend on several factors and nutrition is one of the main ones. We intend to understand the role of maternal diabetes-induced fetal programming and its association with a high-fat diet during lifelong in the female F1 generation focusing on reproductive outcomes and the possible changes in physiological systems during pregnancy as well as the repercussions on the F2 generation at birth. For this, we composed four groups: F1 female pups from control (OC) or from diabetic dams (OD) and fed with standard (SD) or high-fat diet from weaning to full-term pregnancy. During pregnancy, glucose intolerance and insulin sensitivity were evaluated. In a full-term pregnancy, the maternal blood and liver were collected to evaluate redox status markers. The maternal blood, placental tissue, and fetal blood (pool) were collected to evaluate adiponectin and leptin levels. Maternal reproductive parameters were evaluated as well. Maternal diabetes and high-fat diet consumption, in isolation, were both responsible for increased infertility rates and fasting glucose levels in the F1 generation and fetal growth restriction in the F2 generation. The association of both conditions showed, in addition to those, increased lipoperoxidation in maternal erythrocytes, regardless of the increased endogenous antioxidant enzyme activities, glucose intolerance, decreased number of implantation sites and live fetuses, decreased litter, fetal and placental weight, increased preimplantation losses, and increased fetal leptin serum levels. Thus, our findings show that fetal programming caused by maternal diabetes or lifelong high-fat diet consumption leads to similar repercussions in pregnant rats. In addition, the association of both conditions was responsible for glucose intolerance and oxidative stress in the first generation and increased fetal leptin levels in the second generation. Thus, our findings show both the F1 and F2 generations harmed health after maternal hyperglycemic intrauterine environment and exposure to a high-fat diet from weaning until the end of pregnancy.

## 1 Introduction

Diabetes is a complex and chronic disease that requires continuous medical care, with strategies of multifactorial risk reduction and glycemic control (ADA, 2021a). According to the American Diabetes Association (ADA), there are three main classes of *Diabetes mellitus* (DM): type 1 DM (DM1), which is characterized by the autoimmune destruction of pancreatic beta (β)-cells, usually causing insulin deficiency; type 2 DM (DM2) that is characterized by the progressive loss of insulin secretion by β-cells, leading to insulin resistance as a background; and gestational *diabetes mellitus* (GDM) that is diagnosed in the second or third trimester of pregnancy, in which the diabetes is not manifested before pregnancy (ADA, 2021b). The prevalence of diabetes in pregnancy has increased worldwide. In 2019, the International Diabetes Federation (IDF) showed that one in six pregnancies was being affected by hyperglycemia, with 13.6% of pregnancies affected by pre-gestational diabetes and 86.4% affected by GDM. The occurrence of GDM has been described by the International Diabetes Federation as a severe and neglected threat to maternal and child health (IDF, 2019). The hyperglycemic intrauterine environment can interact with the genome of offspring, inducing long-term altered patterns of gene expression and can be transmitted transgenerationally (Gauguier et al., 1990; Boloker et al., 2002). For this reason, the IDF recommends that pregnant women with diabetes or at high risk of developing GDM should monitor their blood glucose to avoid long-term consequences for themselves and transgenerational effects for their children (IDF, 2019).

In addition to maternal glycemic control during pregnancy, adequate nutrition and specific nutrients are important in all periods of life, but they are essential during specific times, such as in intrauterine life and early postnatal life (Greco et al., 2019). Studies have shown that the first 1,000 days after conception (from intrauterine life/pregnancy to the first 2 years of the life of a child) are windows of particular sensibility to environmental factors influencing lifelong trajectories through health and disease (Barker, 2012; Simeoni et al., 2018). In this context, several epidemiologic and experimental studies show that maternal hyperglycemia and an unbalanced diet can induce health consequences several decades after exposure, leading to a higher prevalence of overweight, obesity, DM2, GDM, and reproductive disorders in the adult lives of male and female descendants (Boullu-Ciocca et al., 2008; Glavas et al., 2010; Bouchard et al., 2012; Kayser et al., 2015; Sánches-Guarrido et al., 2015; World Health Organization, 2019). However, the exposure to adverse conditions during intrauterine development is particularly important to female offspring because it can cause physiological changes that have the potential to alter both the reproductive capacity of the first generation and the health of the second generation through changes in the oocyte (Yao et al., 2021). Moreover, the sex of the embryo also plays an important role in determining how an insult might become part of the epigenome and be transmitted to future generations. In the case of female rats, as the entire repertoire of primordial follicles forms during intrauterine and early neonatal phases, the consequences may be more evident in reproductive aspects than in males (Yao et al., 2021).

Environmental factors and lifestyle have a direct influence on developmental programming and may even reverse those (Gluckman et al., 2008). Leptin and adiponectin are hormones that regulate energy balance and insulin sensitivity, playing a critical role in the establishment of this program (Parent et al., 2014; Choi, 2018). Both hormones have a key role in metabolism, maternal–fetal interaction, and metabolic abnormalities which can lead to pregnancy complications and fetal growth changes (Santos et al., 2015). Intrauterine exposure to hyperglycemia leads to the development of both leptin and insulin resistance in the placenta (Greco et al., 2019), and these alterations are related to maternal glycemic levels (Bouchard et al., 2012; Houde et al., 2013). Moreover, animal and human studies show that leptin and insulin resistance act on hypothalamic receptors and appetite circuits, leading to postnatal hyperphagia, decreased satiety, and subsequent development of metabolic syndrome (Block and El-Osta, 2017). Plasma leptin concentrations, in both dams and their offspring, may play a role in linking nutrition and development (Stocker et al., 2007). Stocker et al. (2007) verified that the impaired glucose tolerance in rats from mothers fed a high-fat diet can be prevented by the administration of leptin to their mothers, indicating the maternal leptin levels during pregnancy and lactation can affect the development of energy balance regulatory systems in their offspring.

In an attempt to assess the repercussions of fetal programming and malnutrition and to eliminate the possible confounding factors between these two simultaneous interventions during pregnancy, we first used a pre-gestational diabetes model (F0 generation), and then a high-fat diet (HFD) was offered only to the female offspring (F1 generation) from weaning until full-term pregnancy. Thus, we intend to understand the role of maternal diabetes-induced fetal programming and its association with a high-fat diet during lifelong in the female F1 generation focusing on reproductive outcomes and the possible changes in physiological systems represented by glycemic metabolism, redox status, concentrations of leptin and adiponectin, and insulin sensitivity as well as the repercussions on the F2 generation at birth.

## 2 Methods

### 2.1 Ethics

The Ethics Committee for the Use of Animals of Botucatu Medical School approved all the methods adopted in this study (Protocol CEUA Number: 1218/2017). Animal handling was performed in accordance with the International Guiding Principles for Biomedical Research Involving Animals promulgated by the Society for the Study of Reproduction and with the guidelines provided by the Brazilian College of Animal Experimentation.

### 2.2 Calculation of Sample Size

Based on previous experiments conducted in our laboratory, each rat from a different litter was used for circulating glycemic calculation obtained by area under the curve (AUC) during the oral glucose tolerance test (OGTT), and using 90% power and an error type I of 5%, the effect size was determined as 10 rats per group.

### 2.3 Animals

The animals were maintained at the local laboratory of Botucatu Medical School (Unesp) under controlled conditions of temperature (22 ± 2°C), humidity (50 ± 10%), and light/dark cycle (12 h) in polypropylene cages lined with wood shavings. Filtered water and feed were offered *ad libitum*. As a form of environmental enrichment, paper balls were used in the cages (Simpson and Kelly, 2011).

### 2.4 Experimental Approach

#### 2.4.1 Experimental Approach for Parental Generation (F0)

**FIGURE 1:**
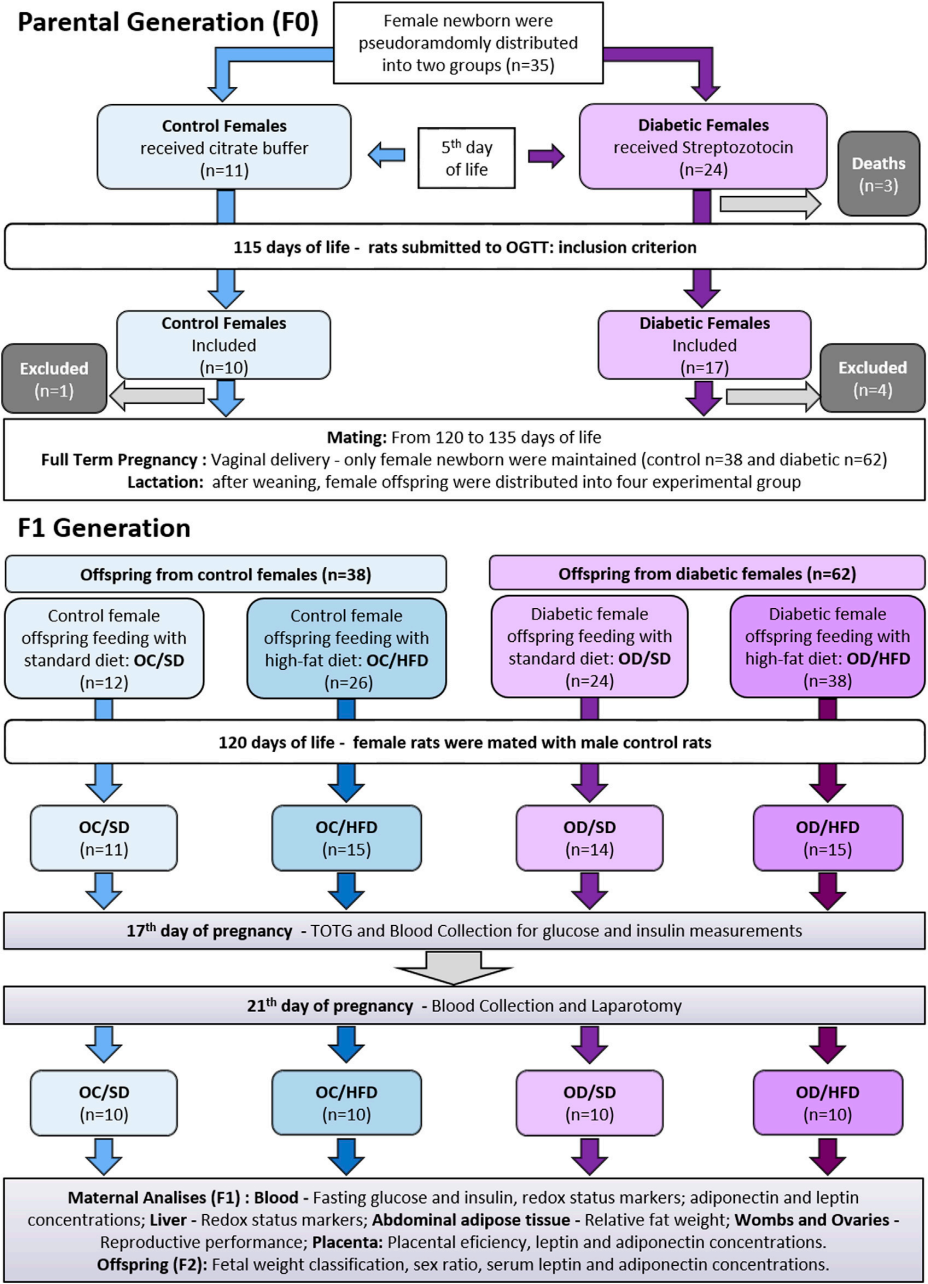
Experimental approach for parental and offspring generation.

The parental generation (F0) aimed to create an inadequate intrauterine environment leading to fetal programming based on the induction of experimental diabetes. The experimental sequence was described in Figure 1.

Male and female Sprague-Dawley rats (200–250 g) were mated overnight to obtain female pups to induce or not induce diabetes during the perinatal period of these offspring. The following morning, when spermatozoa were found in the vaginal smear, was designated as day 0 of pregnancy. The female rats that were not mated after 15 consecutive days were considered infertile and excluded from this study (Moraes-Souza et al., 2017). The pregnant rats were randomly assigned to diabetic (D) and nondiabetic groups by draw, and the female rats were kept in individual polypropylene cages during pregnancy. On day 21 of pregnancy, the rats had a vaginal delivery. Eight pups per dam were kept with their dams until postnatal day (PND) 22, considering the highest number of females wherever possible. We prioritize female rats because our research group studies the transgenerational effects related to pregnancy. The excess pups were euthanized by decapitation (Sinzato et al., 2021).

#### 2.4.2 Diabetes Induction

On postnatal day 5 (PND), the female pups (First Generation—F1) were injected with streptozotocin (Sigma Aldrich^®^, United States, a dose of 70 mg/kg, intraperitoneal route) for diabetes induction, while control female rats received an equivalent volume of vehicle (citrate buffer - 0.01 M pH 4.5), as previously described (Damasceno et al., 2011). Male offspring were maintained with their mothers until weaning and then were euthanized by decapitation or used by other investigators. The blood glucose levels were determined in female adult rats on a PND 75, as established by Gallego et al. (2018—with modifications) and classified by the American Diabetes Association (ADA, 2019). Following, female rats were considered diabetic when presenting glycemia ≥200 mg/dl (11.11 mmol/L) at least at a one-time point during the oral glucose tolerance test (OGTT). In the control group, only rats with glycemia <140 mg/dl (7.77 mmol/L) at least at a three-time point during the OGTT were included. The female rats that were not accomplishing the aforementioned characteristics were anesthetized by using sodium thiopental (Thiopentax^®^, Cristália, Brazil—120 mg/kg dose), euthanized, and excluded from this study.

#### 2.4.3 Mating, Pregnancy, and Lactation

After inclusion criteria on PND 90, the diabetic (D) and nondiabetic (control—C) female rats were mated as previously described. The offspring were obtained through vaginal delivery. To avoid differences in maternal care between male and female pups (Beery and Francis, 2011) which could result in epigenetic consequences (Champagne et al., 2003), after birth, all-male newborns were euthanized by decapitation, while female newborns were kept in a range from six to eight pups per litter until weaning day 22, to maintain the milk consumption balance. The excess number of female pups was also euthanized by decapitation. When the litter had less than six female pups, it was excluded from the study and redirect to another experiment in our group. After weaning, female pups from nondiabetic (control) and diabetic dams were randomly assigned to compose the experimental groups.

Experimental approach for female pups from the maternal diabetic environment (F1 generation).

#### 2.4.4 Experimental Groups and Dietary Patterns

Upon weaning, the female pups from diabetic (OD) and control (OC) mothers were pseudo-randomized by lot respecting a maximum of four female pups per mother (with two sisters to each group: C or D). The other females from the same litter were used in another subproject with non-pregnant rats (Protocol CEUA Number: 1218/2017). The OD and OC groups were further distributed into two other groups according to their diets: standard diet (SD - Kcal content: 28.54% protein, 62.65% carbohydrate, 8.7% fat, Purina^®^, Brazil) or high-fat diet (HFD - Kcal content: 23.43% protein, 46.63% carbohydrate, 30% fat, using lard as the main fat source). Additional bromatological comparison between the diets is shown in Supplementary Table S1. Thus, four experimental groups were established: OC/SD: female pups from control mothers and fed standard diet; OC/HFD: female pups from control mothers and fed high-fat diet; OD/SD: female pups from diabetic mothers and fed standard diet; and OD/HFD: female pups from diabetic mothers and fed high-fat diet. All the groups were followed from weaning to PND 120. The HFD was handmade at our institution, adequately supplemented with vitamins and minerals, and maintained under refrigeration until the time of use (Paula et al., 2021). Given the fact that the diets had visual characteristics that easily allow their distinction, random housing, and blinding of caregivers and/or investigators were not possible.

#### 2.4.5 Mating

From PND 120, diabetic (D) and control (C) female rats were mated as previously described.

#### 2.4.6 Pregnancy

##### 2.4.6.1 Oral Glucose Tolerance Test (OGTT) and QUICK Index.

Maternal weight, water consumption, and food intake (evaluated as gram intake and energy intake) were monitored during the entire pregnancy. Energy intake was determined to observe if the moderate differences showed in body weight evolution would be related to differences in food intake (Pérez-Matute et al., 2007). On pregnancy day (PD) 17, OGTT was performed for glucose tolerance evaluation as previously described (Tai, 1994; Gallego et al., 2019). In this same test, blood samples were collected at fasting for insulin measurements using an ELISA commercial kit (Crystal Chemical^®^ Code: 90060, United States). The quantitative insulin sensitivity check index (QUICK index) was calculated as defined by Katz et al. (2000) as QUICKI = 1/[(log (I_0_) + log (G_0_)], where I_0_ is the fasting plasma insulin level (microunits/mL), and G_0_ is the fasting blood glucose level (milligrams per dL).

##### 2.4.6.2 Blood Collection and Laparotomy

On PD 21, the rats were intraperitoneally anesthetized by using sodium thiopental (Thiopentax^®^, Cristália, Brazil—120 mg/kg dose) and decapitated. The collection of maternal blood was performed to obtain serum and washed erythrocyte samples according to the ELISA kit’s instructions for leptin and adiponectin (#80570; # E-EL-R0582) assays and according to Souza et al. (2010) for redox status analyses, respectively. All samples were stored at −80°C until further analysis. Uterine horns, ovaries, newborns, and placentas were removed and weighed for evaluation of maternal reproductive performance, birth weight classification, and placental efficiency (Sinzato et al., 2021). Visceral fat pads from the abdominal cavity (periovarian, periuterine, perirenal, and retroperitoneal) were pooled and weighed to calculate the relative fat weight (grams of total visceral fat/100 g of body weight). Maternal liver and two placentas from each litter (one from female newborns and the other from one of the male newborns were randomly assigned) were removed and quickly frozen in liquid nitrogen and stored in a freezer at -80°C until homogenates were performed. The liver homogenate (modified from Spada et al., 2014) was used for redox status evaluation, and the placental homogenate (modified from Portela et al., 2021) was used for leptin and adiponectin determinations. Newborns were sexed and all male and female newborns from each litter were euthanized and killed by decapitation for blood collection (pool). Serum samples from each pool were obtained as described for maternal blood and used for leptin and adiponectin determinations.

##### 2.4.6.3 Reproductive Outcomes and Birth Weight Classification

The mothers who presented no live newborns at the end of pregnancy were not included in the reproductive analyses. The ovaries were used to count the number of corpora lutea as an indirect parameter to assess the number of oocytes. The uterus of pregnant rats was dissected to count the number of living and dead fetuses, resorptions (embryonic deaths), and implantation sites (Sinzato et al., 2021). When the implantation sites were visually undetectable, the Salewski (1964) reagent was used as a dye. The percentage of embryonic loss before implantation (preimplantation loss rate), the percentage of embryonic loss after implantation (postimplantation loss rate), and the classification of the birth weight into small for gestational age (SGA), appropriate for gestational age (AGA), and large for gestational age (LGA) were made according to Sinzato et al. (2021).

##### 2.4.6.4 Liver Homogenates for Redox Status Analyses

Liver samples from a maternal organism (F1 generation) frozen samples were homogenized using the bullet blender system, adding zirconium beads, phosphate-buffered saline (PBS—0.1 M Na2HPO4, 0.1 M KH2PO4, 0.1 M EDTA, pH 7.8), and protease inhibitor cocktail (#P8340) for glutathione peroxidase (GSH-Px), catalase (CAT), reduced thiol groups (-SH), and hydrogen peroxide (H_2_O_2_—reactive oxygen species) assays. For thiobarbituric acid reactive substances (TBARS–lipoperoxidation biomarker) assay, RIPA lysis buffer (1X #92590) was used. For superoxide dismutase (SOD), a solution with 20 mM HEPES, 1 mM EGTA, 210 mM mannitol, and 70 mM sacarose (pH 7.2) was used. Then, all samples were centrifuged at 1,600 *x g* for 10 min at 4°C for GSH-Px, CAT, -SH, and H_2_O_2_; 240 *x g* for 10 min at 4°C for TBARS; and 1.500 *x g* for 5 min at 4°C for SOD. After centrifugation, the supernatant was stored in a freezer at -80°C until assays. Protein concentrations were analyzed by the Bradford method (Bradford, 1976).

##### 2.4.6.5 Placental Homogenate for Leptin and Adiponectin Analyses

From here, all procedures were performed with the investigator blinded to the analyses. For the placental homogenate, out of ten samples collected from male newborns, five were randomly assigned to analyses. The same procedure has been made for the placentas from female newborns. Placental frozen samples were homogenized using the bullet blender homogenizer^®^ (Next Advanced, NY, United States) adding zirconium oxide beads (1 mm–Code ZrOB10, and 2 mm–ZrOB20, Next Advanced, NY, United States ) and lysis buffer (RIPA 1X #9806), protease/phosphatase inhibitor cocktail (1X #5872), phenylmethane sulfonyl fluoride (PMSF 1 mM #8553), and the remaining volume completed with water purified by the ultra purifying master system^®^ (GEHAKA, São Paulo, Brazil). After homogenization, the samples were kept and incubated on ice and homogenized by vortex every 15 min for 2 hours, then centrifuged at 7,000 × g for 15 min at 4°C, and the supernatant was stored at -20°C. Protein concentration was analyzed by the Bradford method using the BSA curve as a standard. The final protein concentration in μg/μL was determined based on the BSA standard curve (Bradford, 1976) performed at each dose. Samples were normalized by the lowest protein concentration obtained after measurement.

##### 2.4.6.6 Evaluation of redox status and hormone assays

Samples of washed red blood cells and liver homogenates were used to evaluate the redox status of a maternal organism following the protocols from Yagi (1976) and Buege and Aust (1978) for TBARS; Boyne and Ellman (1972) modified by Hu (1994) for -SH; Noble and Gibson (1970) for H_2_O_2_; Marklund and Marklund (1974) for SOD; Lawrence and Burke (1976) for GSH-Px; and Aebi (1984) for CAT.

Fasting serum insulin was determined using an ultra-sensitivity ELISA kit from Crystal Chemicals^→^, United States (Code: 90060). For maternal, placental, and fetal adiponectin and leptin determinations were used, respectively, an ELISA kit from Crystal Chemicals^®^, United States (Code: 80570) and an ELISA kit from Elabscience^®^, United States (Code: E-EL-R0582).

### 2.5 Statistical Analysis

To calculate the sample size, 10 mothers of the parental generation were used, each one from a different litter, and a completely randomized design was made by the Research Support Office of Botucatu Medical School, Unesp. Approximately 10 animals/groups have been established for each group. For the analysis of the F1 generation, we used a maximum of two female rats from the same litter for the experiments. All newborns from each litter were used in the F2 generation analyses. There were no used repeated measures for any parameter. For asymmetric distribution of the data (fetal and placental weights, glycemia of OGTT and AUC, serum insulin concentrations during OGTT, and TBARS levels), the gamma distribution test followed by the Wald multiple comparison test was used. For the QUICK Index, adiponectin and leptin concentrations, other redox status markers, maternal weight gain, maternal body and litter weight, water intake, food consumption, and energy intake, one-way ANOVA followed by the Tukey multiple comparison test was used. The Poisson distribution test was used for corpora lutea, implantation and alive fetus numbers, and placental efficiency since these parameters presented the asymmetric distribution of the data. For proportion analysis (sex ratio, fetal weight classification, and pre and postimplantation loss percentages), the chi-square test was used. A minimum confidence limit of 95% (*p* < 0.05) was considered statistically significant for all statistical comparisons. All statistical analyses were performed using SAS software for Windows, v.9.4.

## 3 Results

### 3.1 Fertility Rate, Maternal Weight and Water, and Food Consumption

The fertility rates were 91.7%, 57.7%, 58.3%, and 39.5% in OC/SD, OC/HFD, OD/SD, and OD/HFD, respectively. The OC/HFD and OD/SD groups presented no differences compared to the control group (OC/SD) (*p* = 0.060 and *p* = 0.059, respectively). The OD/HFD group presented a lower fertility rate compared to the OC/SD (*p* < 0.05) (Table 1). The percentage of female rats with positive vaginal smear did not reach full-term pregnancy is presented in Table 1 (*p* > 0.05). These animals were not included in the statistical analysis of the preimplantation losses.

**TABLE 1:** Maternal reproductive performance, relative fat weight, placental and birth weight, and placental efficiency on the 21^st^ day of pregnancy from F1 control pregnant offspring (OC) and diabetic pregnant offspring (OD) that received standard diet (SD) or high-fat diet (HFD) from weaning.

Variable	Group			
	OC/SD	OC/HFD	OD/SD	OD/HFD
Fertility rate (%)	91.7	58.3	57.7	39.5*
Non-pregnant at full-term (%)	9.1	33.3	26.7	33.3
Number of corpora lutea	13.9 ± 1.7	14.0 ± 1.6	13.8 ± 0.9	13.2 ± 1.7
Number of implantation	13.7 ± 1.8	12.9 ± 1.5	11.1 ± 4.5	7.1 ± 5.3*^#^
Number of embryonic deaths	1.0 ± 1.2	1.9 ± 2.0	1.4 ± 1.9	0.8 ± 1.3
Number of live fetuses	12.7 ± 1.6	10.9 ± 2.4	9.7 ± 4.9	6.6 ± 5.2*^#^
Preimplantation loss (%)	1.1	7.5	12.5	28.7*
Postimplantation loss (%)	7.0	15.3	18.2	12.1
Maternal weight gain (g)	126.3 ± 31.3	82.1 ± 24.5*	109.3 ± 17.7	88.0 ± 16.5*^$^
Litter weight (g)	99.6 ± 12.8	79.0 ± 16.3	79.5 ± 25.9	58.9 ± 29.8*
Relative fat weight (g/100 g of body weight)	2.49 ± 0.58	4.14 ± 1.02*	3.68 ± 1.43	3.18 ± 0.23
Birth weight (g)	5.84 ± 0.33	5.14 ± 0.66*	5.29 ± 0.68*	4.81 ± 0.87*^#$^
Placental weight (g)	0.56 ± 0.10	0.53 ± 0.08	0.60 ± 0.08*	0.51 ± 0.12^$^
Placental efficiency	10.74 ± 1.43	9.89 ± 1.57*	8.86 ± 1.13*	9.68 ± 2.10*^$^

Values expressed as mean ± standard deviation (SD). *n* = 10 rats/group. *p < 0.05—compared to the OC/SD group; #*p* < 0.05—compared to the OC/HFD group; $*p* < 0.05—compared to the OD/SD group (Tukey Multiple Comparison Test, and Poisson distribution test was used for corpora lutea, implantation and alive fetus numbers, and placental efficiency and chi-square test for proportions).

The maternal weight presented no differences between OC/HFD and OC/SD at PD0, PD_7_, PD_14,_ and PD_21_ compared to other groups. The OD/HFD rats showed an increase in relative fat weight at full-term pregnancy (Table 1) and maternal weight PD_7_ of pregnancy compared to the OC/SD, OC/HFD, and OD/HFD dams (Supplementary Figure S2A). There were no differences in water consumption among groups (Supplementary Figure S2D). The food consumption was lower in OC/HFD rats at PD_14_ and PD_21_ of pregnancy compared to the OC/SD. The OD/HFD group presented lower food intake on day 14 of pregnancy than the OC/SD rats and compared to the OD/SD group at day 21 of pregnancy (Supplementary Figure S2B). However, when we evaluated the energy intake, the total calories ingested (Kcal/day) in the OC/HFD and OD/HFD groups (fed with a high-fat diet) at PD0 and PD_7_ had an increase compared to those in the OC/SD and OD/SD groups. At PD_14_, only the OD/HFD rats presented a higher energy intake on the same day of pregnancy in relation to the OD/SD (Supplementary Figure S2C).

### 3.2 TOTG, AUC, Fasting Insulin, and QUICK Index

**FIGURE 2:**
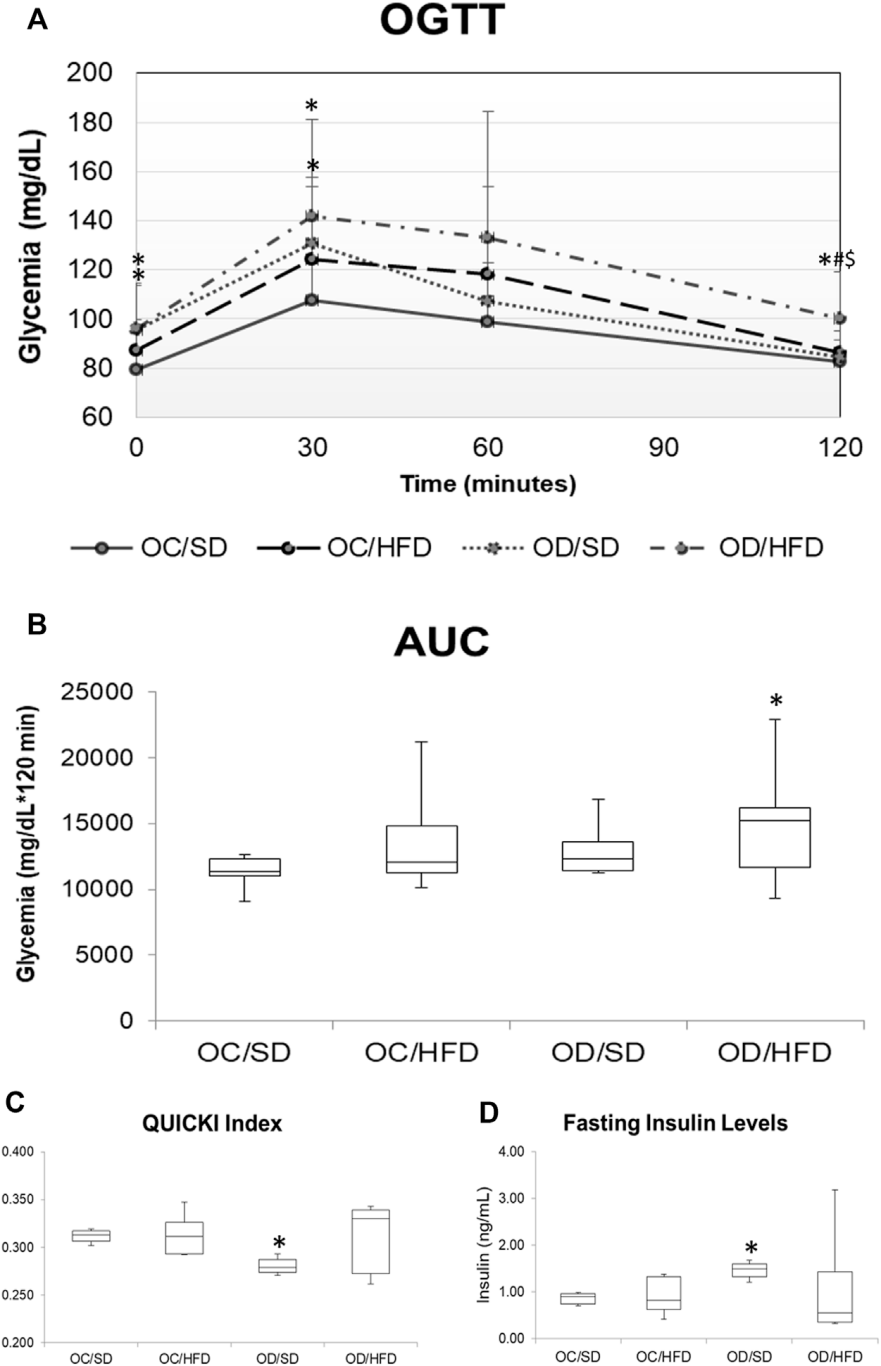
**(A)** OGTT, oral glucose tolerance test; **(B)** AUC, area under the curve; **(C)** QUICK Index; and **(D)** fasting maternal insulin levels on PD17 from OC, control offspring; and OD, diabetic offspring that received a SD, standard diet; or HFD, high-fat diet from weaning. Values are expressed as mean ± SD, standard deviation. *n* = 10 rats/group. *p < 0.05—compared to the OC/SD group; #*p* < 0.05—compared to the OC/HFD group; $*p* < 0.05—compared to the OD/SD group. For OGTT and AUC, the gamma distribution test was used and for QUICK Index, Tukey Multiple Comparison Test was used.

Figure 2 shows the comparison of the glycemic levels by OGTT (Figure 2A) and AUC (Figure 2B), QUICK Index (Figure 2C), and fasting insulin (Figure 2D) among groups. The OD/SD and OD/HFD groups showed increased fasting glucose levels compared to the OC/SD group. At time point 30 of TOTG, all groups had an increase in the blood glucose levels compared to time point 0, but only the OD/SD and OD/HFD groups presented glycemic levels superior to 140 mg/dl. The OC/HFD and OD/HFD groups had increased glycemia compared to the OC/SD and OD/SD rats at 60 min of TOTG. At 120 min of TOTG, the OD/HFD group showed an increase in blood glucose levels compared to the other groups (OC/SD, OC/HFD, and OD/SD). AUC showed increased total glucose during TOTG of OC/HFD, OD/SD, and OD/HFD compared to the OC/SD group. The fasting maternal insulin concentration was increased in the OD/SD and OD/HFD rats compared to the OC/SD group, and the QUICK Index (insulin sensitivity index) was decreased in the OD/SD group compared to the other groups.

### 3.3 Analysis of Redox Status Markers in Maternal Blood and Liver Samples

**FIGURE 3:**
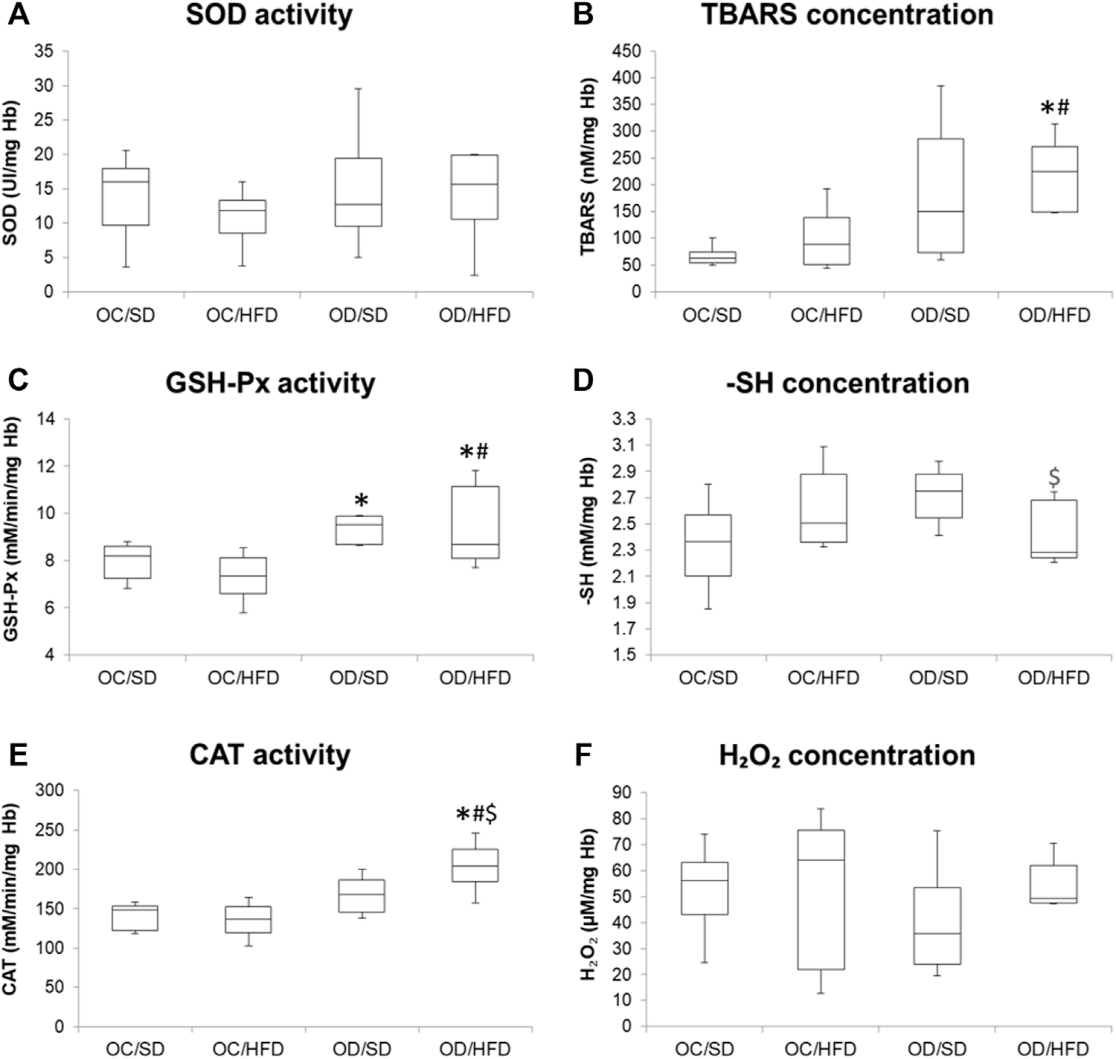
**(A)** SOD, superoxide dismutase activity; **(B)** TBARS, tiobarbituric acid reagent species concentration; **(C)** GSH-Px, glutathione peroxidase activity; **(D)** reduced thiol groups concentration (-SH) **(E)** CAT, catalase activity; and **(F)** hydrogen peroxide concentration (H_2_O_2_) in washed erythrocytes at full-term pregnancy from OC, control offspring; and OD, diabetic offspring that received a SD, standard diet or HFD, high-fat diet; from weaning. Values are expressed as mean ± SD, standard deviation; *n* = 10 rats/group. *p < 0.05—compared to the OC/SD group; #*p* < 0.05—compared to the OC/HFD group; $*p* < 0.05—compared to the OD/SD group (Tukey Multiple Comparison Test).

**FIGURE 4:**
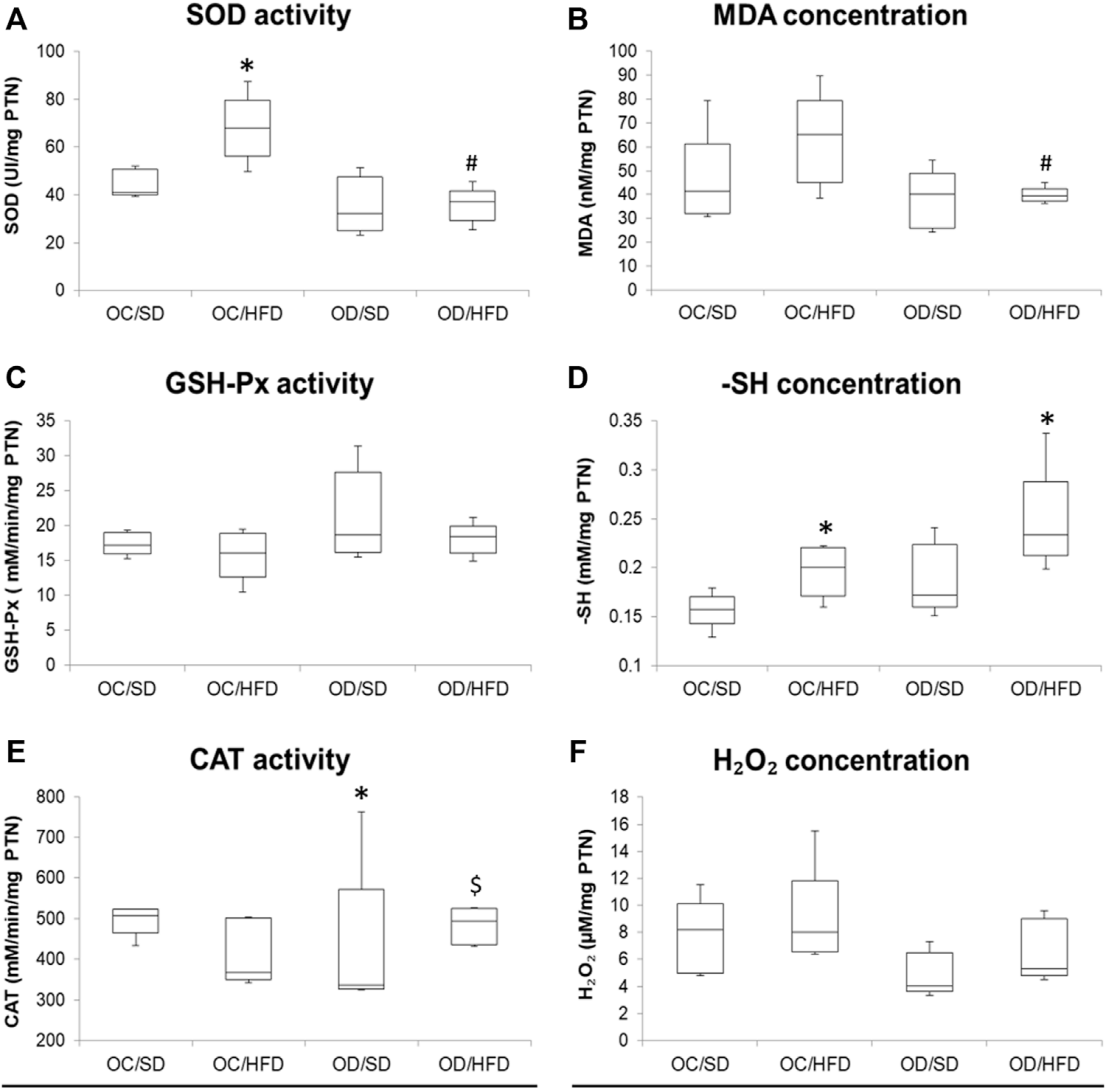
**(A)** SOD, superoxide dismutase activity; **(B)** MDA, malondialdehyde concentration; **(C)** GSH-Px, glutathione peroxidase activity; **(D)** -SH, reduced thiol groups concentration; **(E)** CAT, catalase activity, and **(F)** H_2_O_2_, hydrogen peroxide concentration in maternal liver at full-term pregnancy from OC, control offspring; and OD, diabetic offspring that received a SD, standard diet or HFD, high-fat diet; from weaning. Values are expressed as mean ± SD, standard deviation; *n* = 10 rats/group. *p < 0.05—compared to the OC/SD group; #*p* < 0.05—compared to the OC/HFD group; $*p* < 0.05—compared to the OD/SD group (Tukey Multiple Comparison Test).

Figures 3, 4 show, respectively, redox status markers sampled from maternal washed erythrocytes and liver. There was no difference in SOD activity, H_2_O_2,_ and -SH concentrations among groups in maternal washed erythrocytes. There was an increase in TBARS concentration in OD/HFD dams compared to the OC/SD and OC/HFD dams. GSH-Px activities were increased in OD/SD dams compared to control dams and in OD/HFD dams compared to OC/SD and OC/HFD dams. CAT activity was increased in the OD/HFD group compared to the other groups.

Considering maternal liver samples, there were no differences in H_2_O_2_ concentrations, GSH-Px, and CAT activities among groups. MDA concentration was decreased in OD/HFD compared to OC/HFD rats. There was an increased SOD activity in OC/HFD dams compared to OC/SD dams and a decreased SOD activity in the OD/HFD rats compared to OC/HFD rats. Increased -SH levels were observed in the OC/HFD and OD/HFD rats when compared to the control group.

### 3.4 Adiponectin and Leptin Concentrations From Maternal and Fetal Serum and Placental Homogenate

**FIGURE 5:**
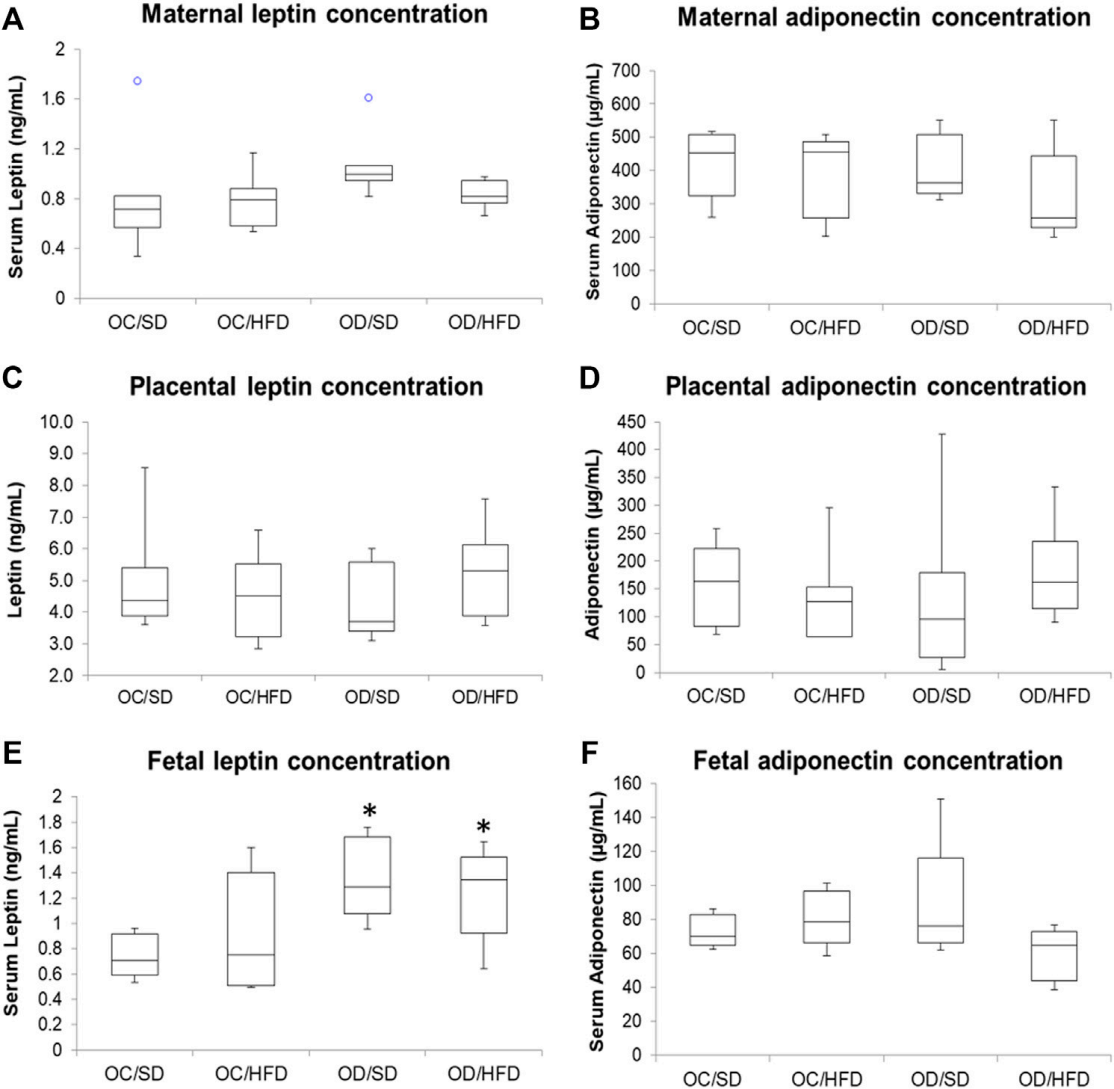
**(A)** Maternal serum leptin; **(B)** maternal serum adiponectin; **(C)** placental leptin; **(D)** placental adiponectin; **(E)** fetal serum leptin; and **(F)** fetal serum adiponectin concentrations from control offspring (OC) and diabetic offspring (OD) that received a standard diet (SD) or high-fat diet (HFD) from weaning. Values are expressed as mean ± standard deviation (SD). n = 10 rats/group. *p < 0.05—compared to the OC/SD group (Tukey Multiple Comparison Test).° Purple dots represent the outliers of the respective groups.

Figure 5 shows, respectively, leptin and adiponectin concentrations in maternal (A and B) and fetal (E and F) serum and placental homogenates (C and D) of rats at term pregnancy. As there was no difference between male and female rats, we chose to present the data together. The adiponectin levels showed no differences in the different tissues among the groups. The leptin concentrations of the maternal serum and placental homogenate presented no difference among groups. There was an increase in serum leptin concentration in the fetuses from OD/SD and OD/HFD dams compared to the OC/SD rats.

### 3.5 Maternal Reproductive Performance, Sex Ratio, and Fetal Weight Classification

Table 1 shows the fertility rate, percentage of non-pregnant rats, reproductive performance, relative fat weight, placental and fetal weight, and placental efficiency of the rats. There were no significant differences in the number of corpora lutea, postimplantation loss percentage, and maternal weight gain among groups. The OC/HFD and OD/SD dams showed reduced litter and fetal weight compared to the OC/SD group. The relative fat weight was increased in the OC/HFD rats than that in the OC/SD dams. The OD/SD group showed lower fetal weight and placental efficiency, and a greater placental weight compared to OC/SD rats. The placental weight and percentage of preimplantation losses were also decreased in OC/HFD and OD/SD in relation to OC/SD. The OD/HFD presented a decrease in the number of implantations, live fetuses, litter, and weight, and increased preimplantation loss percentage compared to the OC/SD, OC/HFD, and OD/SD groups. In addition, the OD/HFD rats showed lower placental weight compared to the OC/SD and OD/SD (Table 1).

**FIGURE 6:**
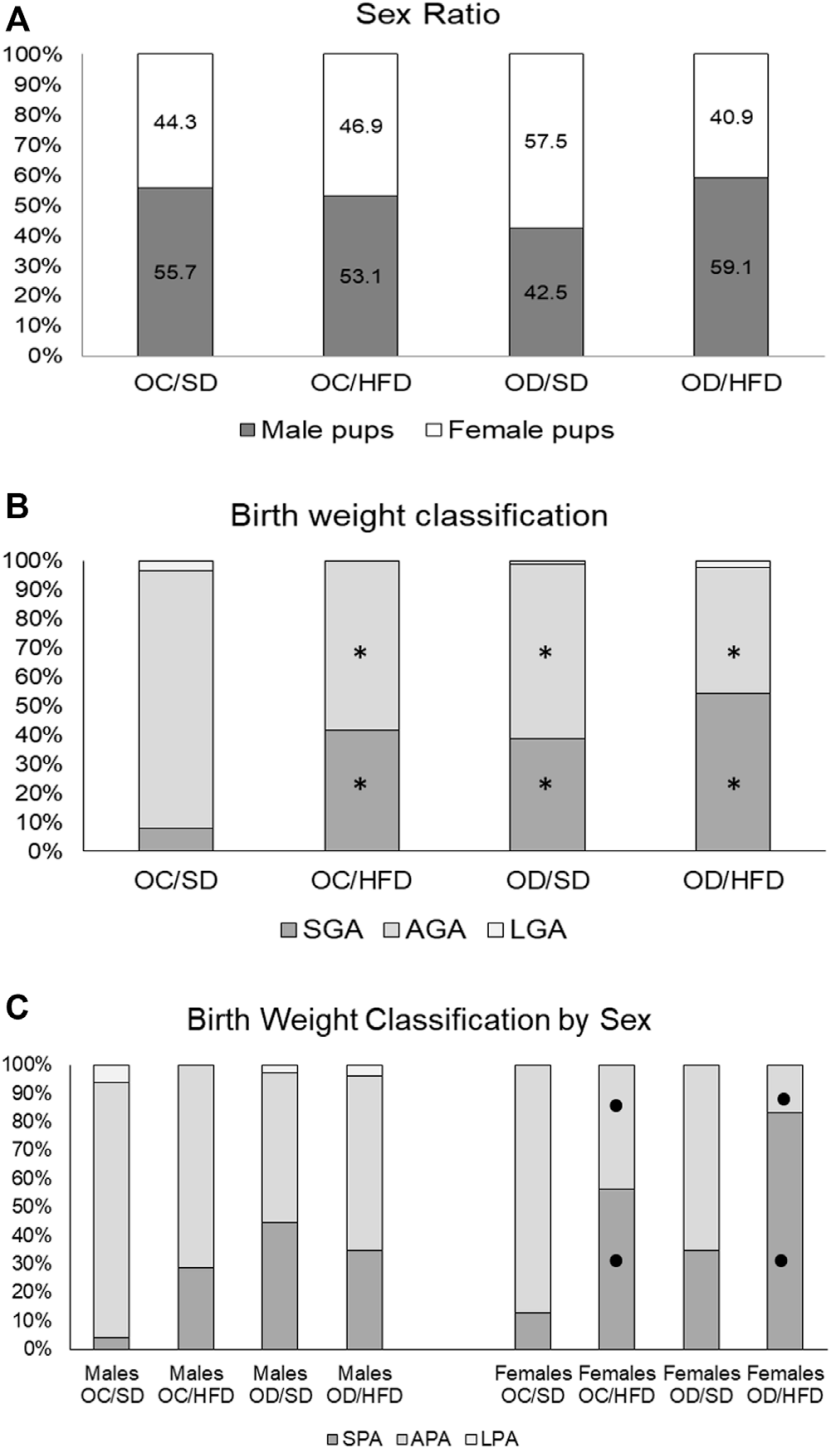
**(A)** Sex ratio, **(B)** birth weight classification, and **(C)** birth weight classification by sex from OC, control offspring; and OD, diabetic offspring that received SD, standard diet or HFD, high-fat diet from weaning. Values are expressed in percentages. Legend: SPA—small fetuses for gestational age, APA—adequate fetuses for gestational age, and LPA—large fetuses for gestational age. *p < 0.05—compared to OC/SD group (Chi-square test) and •*p* < 0.05—between male and female pups from the same group (Chi-square test).

The fetal weight classification showed an increased number of fetuses classified as small for gestational age (SGA) and a decreased number of fetuses classified as adequate for gestational age (AGA) in all groups when compared to the OC/SD (Figure 6). When we compared the fetal weight classification by sex, we observed an increased ratio of SGA and a decreased ratio of AGA in the female pups from OC/HFD and OD/HFD when compared to male pups from the same group. There was no difference in the male and female ratio between the groups and among groups (Figure 6).

## 4 Discussion

Clinical and experimental studies have been performed to evaluate diabetes-induced repercussions (Metzger et al., 2009; Tam et al., 2017; Lowe et al., 2019; Bueno et al., 2020) and/or malnutrition on descendants (Zhang et al., 2008; Mdaki et al., 2016; Tellechea et al., 2017; Hsu & Tain, 2019; Castro-Rodríguez et al., 2020). However, studies addressing the influence of postnatal malnutrition on female offspring that were programmed by intrauterine hyperglycemia are still lacking. Therefore, this study used a rat model with maternal diabetes to reproduce the transgenerational effects of hyperglycemia on female offspring (F1 generation) fed with a high-fat diet from weaning to full-term pregnancy (∼ PND 150). We found that maternal-diabetes fetal programming associated with high-fat diet consumption, was responsible for increased infertility rates, increased lipoperoxidation in maternal erythrocytes, glucose intolerance, decreased number of implantation sites and live fetuses, decreased litter, fetal and placental weight and increased preimplantation losses in the F1 generation, and increased fetal leptin serum levels and fetal growth restriction in the F2 generation.

The increased infertility rate in OD/HFD rats, in our study, may be related to the beginning of oxidative stress status verified in maternal erythrocytes, characterized by higher antioxidative enzyme activities (CAT and GSH-Px) in maternal erythrocytes of OD/HFD rats and an increased TBARS indicating lipoperoxidation. Oxidative stress is related to hyperglycemia, and it is also one of the main causes of alterations in the reproductive systems of women (Agarwal et al., 2012). Previous studies have reported that the balance between reactive oxygen species (ROS) and antioxidants greatly influences different phases of the reproductive activities in female mammalian animals (Al-Gubory et al., 2010; Wang et al., 2017), and oxidative stress conditions may compromise the reproduction and fertility (Agarwal & Allamaneni, 2004; Agarwal et al., 2012). The beginning of oxidative stress status in maternal erythrocytes can be a signal that organs, like the pancreas and ovaries, also present with an unbalanced redox system, and initial changes in this redox status are seen in the red blood cells of OD/HFD rats in our study might be related to the lower fertility and implantation rates as well. Unlike the liver, which presented the high activity of antioxidative enzymes and no alterations in MDA levels in our study, redox status during pregnancy varies among different organs and depends on the local regulation. The pancreas has a lower antioxidant defense and, consequently, a greater susceptibility of beta (β)-cells to oxidative stress (Lenzen et al., 1996). Thus, the maladaptation of the endocrine pancreas to pregnancy can affect maternal glucose and insulin concentrations as seen in OD/HFD rats and harm reproductive organs. Exposure to adverse intrauterine conditions can lead to permanent changes in the structure and function of major physiological systems in developing fetuses (Yao et al., 2021). In rodents, hyperglycemia interferes with the development of fetal female gonads by reducing the weight, diameter, and volume of the ovaries and reducing the number of ovarian follicles and follicular diameter in these offspring (Khaksar et al., 2013). Similarly, overnutrition correlates with poor oocyte development in humans (Larsen, 2015), and a high-fat diet impairs oocyte quality and embryo development in mice (Bermejo-Alvarez et al., 2012). However, to understand the possible mechanisms involved in decreased fertility rates observed in OD/HFD female rats, a study focused on morphologic and molecular alterations in the ovaries of these offspring is underway in our research group.

Despite low fertility rates, when considering only the female rats that reached full-term pregnancy, the indirect parameter evaluated to study the delivered oocyte number–—the number of corpora lutea—presented no difference among experimental groups, showing no ovulation changes in this period. Nevertheless, OD/HFD rats showed a decrease in the number of implantations and live fetuses and higher rates of embryonic losses before implantation. Studies with early embryos (from two cells to blastocyst) in culture with high mean glucose concentration (Fraser et al., 2007) or *in vivo* study using hyperglycemic mother rats to evaluate their pre-embryos (Bueno et al., 2014) showed damages caused by hyperglycemic exposure during early embryonic development. This led to reduced cell numbers and increased apoptosis rates, which caused developmental delay and affected the success of embryonic implantation. Then, the lower number of implantations and live fetuses confirmed in the OD/HFD group might be related to embryonic complications in the early development, leading to higher percentages of preimplantation losses, which contributed to lower litter weight, compromising especially the female fetus growth.

Our results about glycemic metabolism surprisingly showed that the OD/HFD pregnant group presented no difference in insulin sensitivity, but the OD/SD rats had a decreased insulin sensitivity, being both groups performed by the quantitative insulin sensitivity check index (QUICKI). According to Katz et al. (2000), QUICKI was developed as an alternative method for glucose clamps. This method presented the best overall linear correlation with the gold standard clamp measurement, and it was more precise than the homeostasis model assessment of insulin resistance (HOMA-IR). Nevertheless, the authors disclose that QUICKI has limitations as the difficulty in applying it to type 1 diabetic individuals (without endogenous insulin secretion) or in patients with type 1 or type 2 decompensated diabetes (Katz et al., 2000). Considering the diabetic offspring, the OD/SD and OD/HFD rats had higher fasting glucose levels and AUC, confirming intolerance glucose status and higher insulin concentrations, confirming insulin resistance. Another study from our research group with control virgin female pups (OC) and diabetic offspring (OD) that received a standard diet (SD) or high-fat diet (HFD) from weaning until 120 days of life showed the presence of glucose intolerance in the OC/HFD, OD/SD, and OD/HFD rats, an increase in insulin synthesis, and the presence of insulin resistance before pregnancy on OC/HFD and OD/SD rats (Paula et al., 2021). The apparent conflicting result might indicate that these animals possibly maintained an adequate adaptive pancreatic response in pregnancy, ameliorating the glucose intolerance presented before pregnancy. Normal pregnancy is associated with increased insulin resistance as a metabolic adaptation to the nutritional demands of the placenta and fetus. Insulin resistance is normally compensated by an adaptive increase in pancreatic β-cell mass together with enhanced glucose-stimulated insulin release (Moyce and Dolinsky, 2018). Studies have shown an increase in the number of cells and/or size of the pancreatic islet to compensate for the physiological demands of pregnancy (Fujimoto and Polonsky, 2009). The increased β-cell mass depends on a combination of the proliferation of existing β-cells, hypertrophic expansion, differentiation of resident progenitor β-cells, or islet cell transdifferentiation accompanied by a temporary decrease in apoptosis (Butler et al., 2010; Ernst et al., 2011; Bonner-Weir et al., 2012; Moyce and Dolinsky, 2018; Szlapinski and Hill, 2021). Thus, an overview of our results suggests the loss of an adequate adaptation of β-cells in OD/HFD rats during pregnancy, suggesting that QUICKI was not a good index in these cases.

The crosstalk among the maternal pancreas, placenta, and peripheral tissues is essential, and adiponectin and leptin are hormones involved in this signaling. The intrauterine exposure to hyperglycemia and placental methylation of leptin and adiponectin might lead to the development of leptin and insulin resistance and alter appetite circuits leading to postnatal hyperphagia, decreased satiety, and subsequent development of metabolic syndrome (Greco et al., 2019). However, our results showed no alterations in leptin concentration and food consumption related to total energy intake, showing no alterations in these circuits. The data about body weight gain and energy intake are controversial in several studies using different types of high-fat diets. According to the high-fat diet characteristics, overeating is induced, which may work together to promote the storage of dietary fat (Schack-Nielsen et al., 2010; Hull et al., 2011; Donovan et al., 2013). Besides, only female pups coming from control dams fed with a high-fat diet showed higher relative fat weight at term pregnancy, but it had no influence on adiponectin and leptin levels in maternal serum in the OC/HFD, OD/SD, and OD/HFD, corroborating Desai et al. (2014). There were no reported changes in the leptin and adiponectin concentrations measured in the placental tissue. Despite these results, the fetuses from OD/SD and OD/HFD rats had increased leptin serum levels, and these data do not corroborate the literature. Clinical studies have been addressing that leptin levels in the umbilical cord and newborns are positively correlated to birth weight and adiposity (Misra & Trudeau, 2011; Misra et al., 2013; Josefson et al., 2014; Ökdemir et al., 2018; Stefaniak et al., 2019). However, our results showed lower birth weight (SGA fetuses) and hyperleptinemia in the second generation of diabetic rats, which was not what we expected. These conflicting data observed in our rat model can be related to the low-fat percentage at birth in these newborns. Leptin plays an important role in controlling satiety and is an indicator of body fat mass. It is important to consider that this peptide may be involved with fetal growth in the third trimester when human fetal fat is deposited (Desai et al., 2014). Thus, in humans, the newborn fat depot occurs in the intrauterine milieu, however, in rats, this takes place after birth (Herrera and Amusquivar, 2000), which might have influenced the lower birth weight in our laboratory animals in this experiment. Additionally, higher serum leptin levels at birth should predispose to increased blood pressure in adult rats since leptin is well known to be a relevant marker and mediator of vascular dysfunction and hypertension (Gonzalez et al., 2013; Schinzari et al., 2013).

The classification of birth weight showed a significant increase in the proportion of small fetuses for gestational age and a decrease in the proportion of fetuses with adequate weight for gestational age in all groups when compared to control. In humans, high maternal body mass index (BMI) is associated with fetal overgrowth and macrosomia, and an increased risk of premature birth, instead maternal underweight increases the risk of low birth weight and small fetuses for gestational age (Liu et al., 2019). Regardless of this controversial finding, adequate placental function is directly responsible for fetal growth. Our study verified impaired placental efficiency in OD/HFD rats. The placenta plays a role underlying fetal programming and, therefore, is in part related to the origin of the development of health and disease (DOHaD). Both maternal glucose and nutrients from the high-fat diet are transmitted from the maternal to the fetal compartment through the placenta. This dynamic process might lead to changes in long-term health and disease outcomes in the first generation. In addition, the second generation might also be affected and may undergo reprogramming during the embryonic period in the primordial germ cells, which contributes to genetic and epigenetic information in the second generation (Dunn et al., 2011).

Our study showed that fetal restriction was predominant in female fetuses from both groups of dams fed a high-fat diet. The sex of the embryo affects the size of both the fetus and the placenta and the ability of the placenta to respond to adverse stimuli (Clifton, 2010; Mao et al., 2010; Gabory et al., 2013). In our study, this suggests that a high-fat diet interferes differently with male and female placental tissue, and thus, the differences in how male and female placentas cope with stressful conditions indicate that this tissue should also be taken into account if we want to understand how it contributes to health and disease later in life.

In conclusion, our findings show that fetal programming caused by maternal diabetes or lifelong high-fat diet consumption, in isolation, leads to similar repercussions in pregnant rats. In addition, the association of both conditions was responsible for glucose intolerance and oxidative stress in the first generation and increased fetal leptin levels in the second generation. Thus, our findings show both the F1 and F2 generations were negatively affected after the maternal hyperglycemic intrauterine environment and exposure to a high-fat diet from weaning until the end of pregnancy.

## Data Availability

The raw data supporting the conclusion of this article will be made available by the authors, without undue reservation.
