# Supplementary material for: Maternal Diabetes and Postnatal High-Fat Diet on Pregnant Offspring
Source: Front Cell Dev Biol. 2022 May 30;10:818621. doi: 10.3389/fcell.2022.818621 (PMC9189289; doi:10.3389/fcell.2022.818621)
Supplement: Supplementary file 1 [file Table1.docx]

| **Information** | **Food** | |
| --- | --- | --- |
|  | **Standard diet** | **High-fat diet** |
| Crude Energy (kcal/g) | 3.09 | 4.26 |
| Moisture (%/100 g) | 7.45 | 3.65 |
| Dry matter (%/100 g) | 92.55 | 96.35 |
| Mineral Matter (% dry matter) | 6.66 | 2.84 |
| Crude protein (% dry matter) | 25.76 | 26.77 |
| Ether Extract (% dry matter) | 3.49 | 15.19 |
| Crude Fiber (% dry matter) | 43.63 | 45.42 |

**Chart 1_Supplementary data.** Composition of experimental food.

**Figure 1_Supplementary data.** **(A)** Maternal body weight, **(B)** Water consumption **(C)** Food consumption **(D)** Energy intake from control offspring (OC) and diabetic offspring (OD) that received standard diet (SD) or high-fat diet (HFD) from weaning. Values expressed as mean ± standard deviation (SD). n = 10 rats / group. *p<0.05 – compared to the OC/SD group; $p<0.05 – compared to the OD/SD group (Student's t-test). ° Purple dots represent the outliers represent the outliers of the respective groups.
