# Supplementary figures and images for: Maternal Diabetes and Postnatal High-Fat Diet on Pregnant Offspring
Source: Front Cell Dev Biol. 2022 May 30;10:818621. doi: 10.3389/fcell.2022.818621 (PMC9189289; doi:10.3389/fcell.2022.818621)

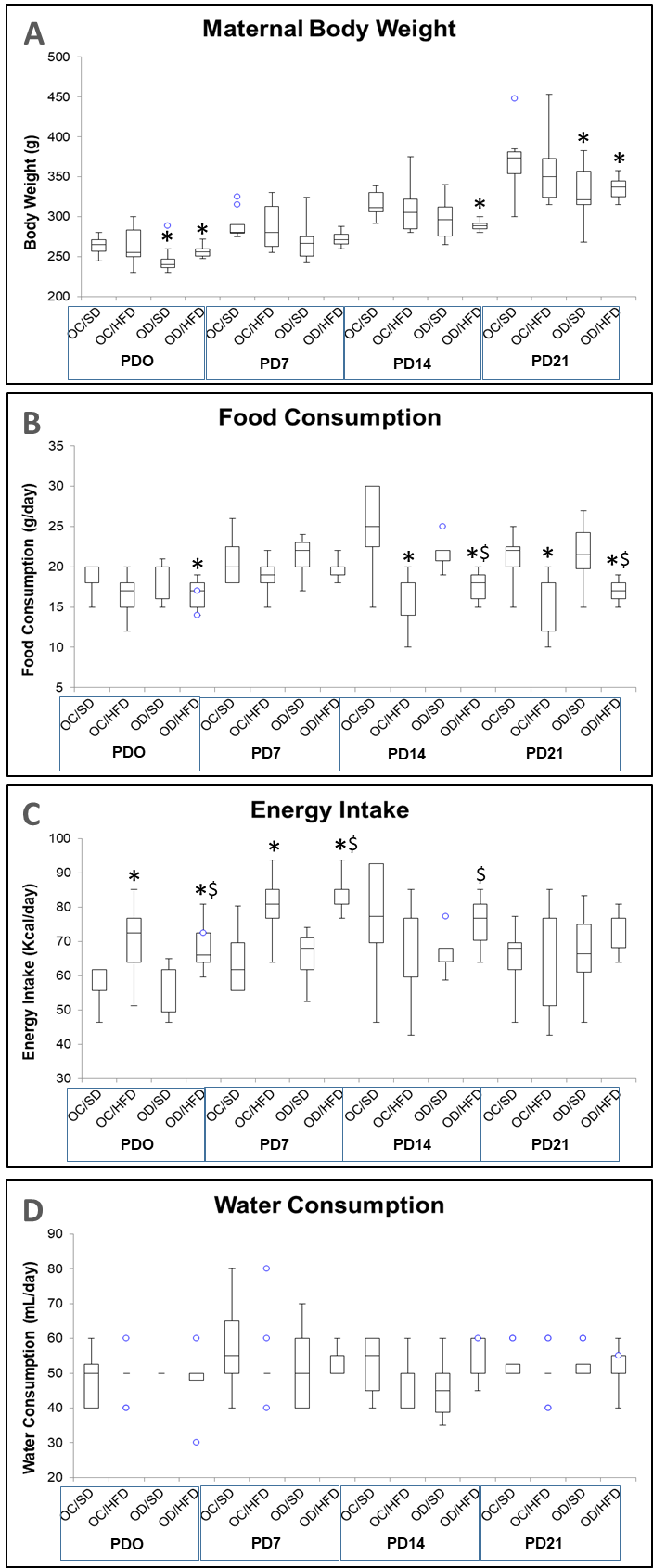

Supplement: Supplementary file 2 [file Image1.tif]
